# FK506 Attenuates the MRP1-Mediated Chemoresistant Phenotype in Glioblastoma Stem-Like Cells

**DOI:** 10.3390/ijms19092697

**Published:** 2018-09-11

**Authors:** Ángelo Torres, Valentina Arriagada, José Ignacio Erices, María de los Ángeles Toro, José Dellis Rocha, Ignacio Niechi, Cristian Carrasco, Carlos Oyarzún, Claudia Quezada

**Affiliations:** 1 Laboratorio de Patología Molecular, Instituto de Bioquímica y Microbiología, Facultad de Ciencias, Universidad Austral de Chile, Valdivia 5090000, Chile; angelo.uach.2018@gmail.com (Á.T.); v.arriagnoack@gmail.com (V.A.); ignacioern@gmail.com (J.I.E.); maria.dl.angeles.tb@hotmail.com (M.d.l.Á.T.); jdellis.rocha@gmail.com (J.D.R.); ignacio.niechi@gmail.com (I.N.); carlosoyarzun@uach.cl (C.O.); 2 Departamento de Patología del Hospital Base de Valdivia (HBV), Valdivia 5090000, Chile; cristian.carrascohv@redsalud.gov.cl

**Keywords:** ATP-binding cassette transporter, glioblastoma stem-like cells, multiple drug resistance, multidrug resistance-associated protein 1, tacrolimus

## Abstract

Poor response to current treatments for glioblastoma has been attributed to the presence of glioblastoma stem-like cells (GSCs). GSCs are able to expel antitumor drugs to the extracellular medium using the multidrug resistance-associated protein 1 (MRP1) transporter. Tacrolimus (FK506) has been identified as an MRP1 regulator in differentiated glioblastoma (GBM) cells (non-GSCs); however, the effect of FK506 on GSCs is currently unknown. The objective of the following research is to evaluate the effect of FK506 on the MRP1-related chemo-resistant phenotype of GSCs. For this, U87MG and C6 glioma cell lines were used to generate non-GSCs and GSCs. mRNA and MRP1-positive cells were evaluated by RT-qPCR and flow cytometry, respectively. A Carboxyfluorescein Diacetate (CFDA)-retention assay was performed to evaluate the MRP1 activity. Apoptosis and MTT assays were employed to evaluate the cytotoxic effects of FK506 plus Vincristine (MRP1 substrate). GSC-derived subcutaneous tumors were generated to evaluate the in vivo effect of FK506/Vincristine treatment. No differences in transcript levels and positive cells for MRP1 were observed in FK506-treated cells. Lesser cell viability, increased apoptosis, and CFDA-retention in the FK506/Vincristine-treated cells were observed. In vivo, the FK506/Vincristine treatment decreased the tumor size as well as ki67, Glial Fibrillary Acidic Protein (GFAP), and nestin expression. We conclude that FK506 confers a chemo-sensitive phenotype to MRP1-drug substrate in GSCs.

## 1. Introduction

Glioblastoma (GBM) is the most common type of brain tumor with the worst prognosis [1,2], which is mainly due to the poor response to the available therapies [3,4,5]. Currently these treatments involve a trimodal therapy, which consists of surgical resection of the tumor followed by radio- and chemotherapy [5,6]. However, patients subjected to these treatments do not exceed, on average, ~15 months of survival [5]. At the same time, the incorporation of new strategies such as anti-proliferative, anti-angiogenic, and immunological therapies have not achieved a great improvement in survival rates [5,7,8,9], so the search for new therapeutic alternatives for GBM has become highly relevant in recent years [5,10]. Studies from ours and other laboratories have found that the main factor that leads to the failure of chemotherapy in GBM is the expression and activity of ATP binding cassette (ABC) transporters [3,11,12]. One of the most relevant and mostly studied of these is multidrug resistance-associated protein 1 (MRP1) [5,12], which is able to extrude several antitumoral drugs to the extracellular medium [11,13], promoting the multiple drug resistance (MDR) phenotype to GBM cells [3,14,15]. In turn, recent work has shown that a cell subpopulation known as glioblastoma stem-like cells (GSCs) presents a greater expression and activity of MRP1 and has been proposed as the principal population responsible for the generation and maintenance of the extreme chemo-resistance phenotype in GBM [3,16,17]. Because of this, the search for new molecules that modulate the expression and activity of MRP1, especially in GSCs, has emerged in recent decades [5,18]. One of the molecules identified as a chemo-sensitizing agent is the immunosuppressive drug tacrolimus (FK506), which at subtherapeutic concentrations (15 ng/mL) decreases both the expression and activity of MRP1 in GBM differentiated cells (non-GSCs), promoting chemo-sensitization to the antitumoral drug Vincristine (Vc) [19]. However, the effect of FK506 on the expression and activity of MRP1 in GSCs has not been reported. Therefore, the aim of this study was to evaluate the effect of FK506 in subtherapeutic concentrations on the chemo-resistant MRP1-associated phenotype in GSCs using in vitro and in vivo assays.

## 2. Results

### 2.1. FK506 Decrease MRP1 Activity in Glioblastoma Stem-Like Cells

**Figure 1:**
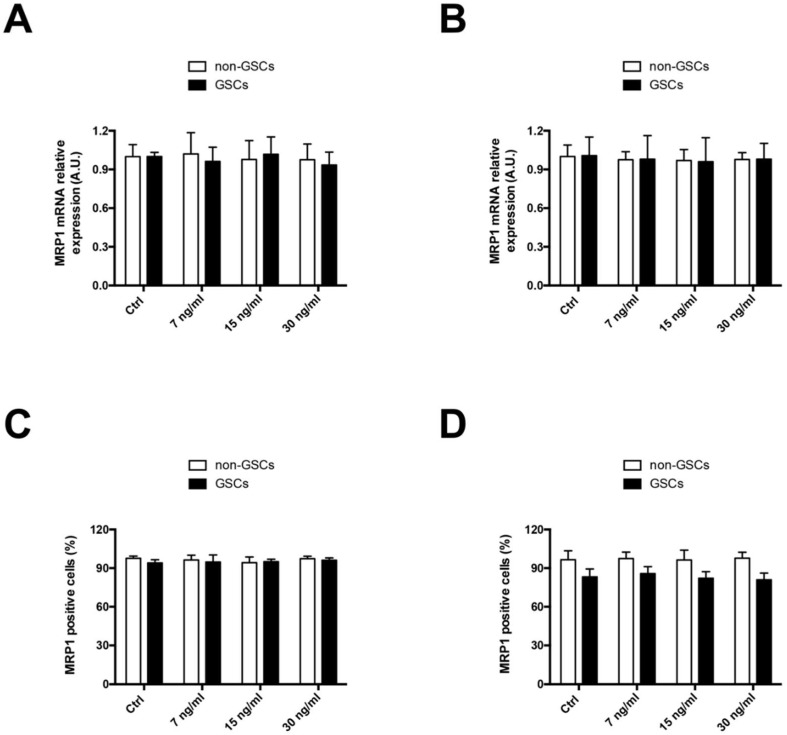
Effect of Tacrolimus (FK506) on multidrug resistance-associated protein 1 (MRP1) levels in U87MG and C6 cells. Cells were treated with FK506 (7, 15, and 30 ng/mL) for 24 h. MRP1 mRNA levels were measured by RT-qPCR in U87MG cells (**A**) and C6 cells (**B**). Graphs represent the values normalized by actin beta (ACTB). MRP1-positive cells were quantified by flow cytometry in U87MG cells (**C**) and C6 cells (**D**). White bars represent non-glioblastoma stem-like cells (GSCs) and black bars represent glioblastoma stem-like cells (GSCs).

The effect of FK506 on in vitro MRP1 expression was evaluated in non-GSCs and GSCs of U87MG (human glioblastoma) and C6 (rat glioma) cell lines. Cells were incubated for 24 h with FK506 at concentrations of 7, 15, and 30 ng/mL. No differences were observed in MRP1 mRNA transcript levels under FK506 treatment in both non-GSCs and GSCs of U87MG (Figure 1A) and C6 (Figure 1B). Similarly, no differences were observed in MRP1-positive cells under FK506 treatment in non-GSCs and GSCs of U87MG (Figure 1C) and C6 (Figure 1D) cell lines.

**Figure 2:**
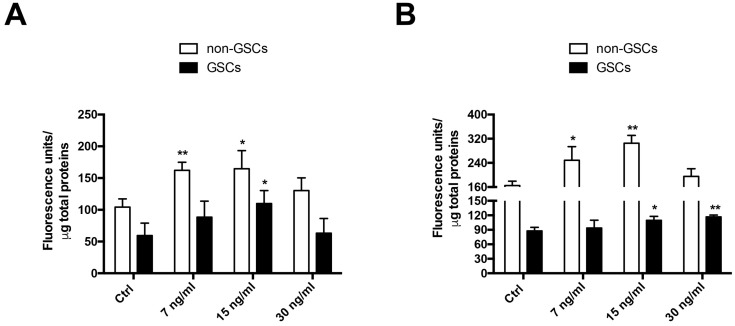
Effect of FK506 on MRP1 activity in U87MG and C6 cells. Cells were treated with FK506 (7, 15, and 30 ng/mL) for 24 h. Activity was measured by MRP1 fluorescent substrate Carboxyfluorescein Diacetate (CFDA) accumulation. The graphs represent fluorescence units normalized by protein concentration in U87MG (**A**) and C6 (**B**) cells. White bars represent non-GSCs and black bars represent GSCs. Graphs represent the mean ± S.D. * *p* < 0.05 and ** *p* < 0.01 versus the control condition (Ctrl). *n* = 6.

To evaluate the effect of FK506 on MRP1 activity in U87MG and C6 cell lines, the intracellular accumulation of a fluorescent substrate of MRP1 Carboxyfluorescein Diacetate (CFDA) was measured [3,19]. MRP1 activity in GSCs was ~2-fold higher than that in non-GSCs, in both U87MG and C6 cell lines (Figure 2A,B). MRP1 activity decreased up to 33% and 27% in U87MG non-GSCs treated with FK506 at concentrations of 7 and 15 ng/mL, respectively (Figure 2A). In U87MG GSCs, with 15 ng/mL FK506, MRP1 activity decreased up to 53% (Figure 2A). In non-GSCs C6 cells, MRP1 activity decreased at 7 and 15 ng/mL FK506 in 50% and 84%, respectively (Figure 2B). C6 GSCs treated with 15 and 30 ng/mL FK506 decreased MRP1 activity in 25% and 33%, respectively (Figure 2B). Taken together, these results demonstrate that FK506 decreases MRP1 activity in both non-GSCs and GSCs, suggesting that the chemo-sensitizing effect of this drug is due to changes in the activity of the transporter and not its expression.

### 2.2. FK506 Promotes Apoptosis and MRP1-Dependent Chemo-Sensitization to Vincristine in GSCs

**Figure 3:**
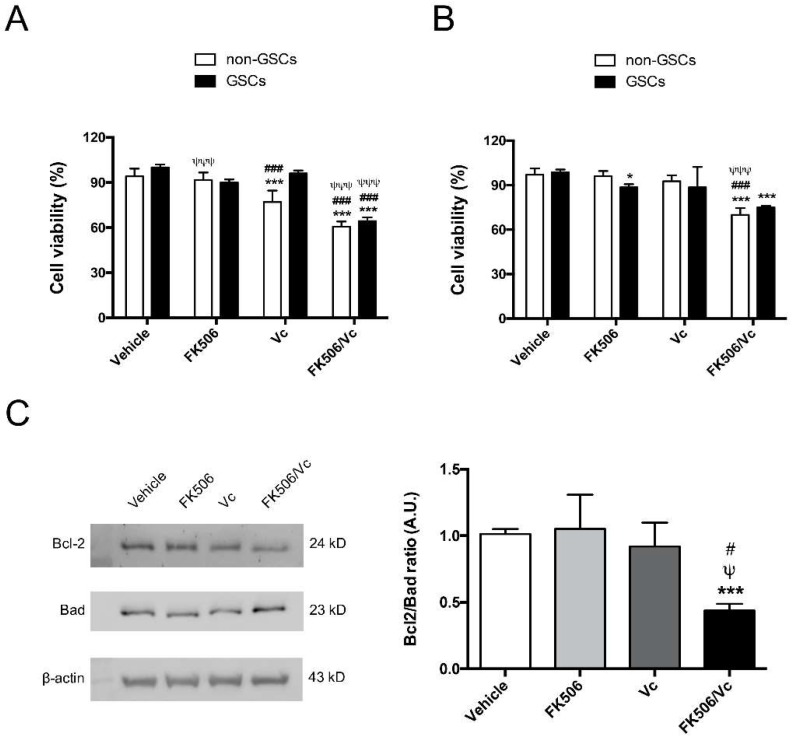

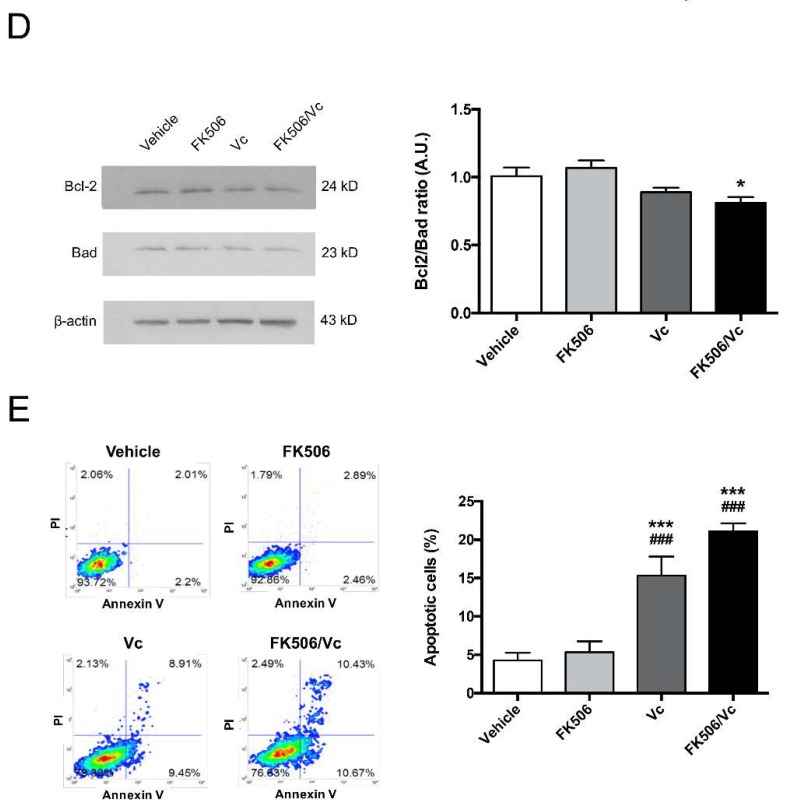
Vincristine in co-treatment with FK506 decreases cell viability by inducing apoptosis in U87MG and C6 cell lines. Cells were treated with FK506 (15 ng/mL) and/or Vincristine (Vc; 0,1 µM) for 24 h. Cell viability was measured by MTT assay in U87MG cells (**A**) and C6 cells (**B**). White bars represent non-GSCs and black bars represent GSCs. Apoptosis was measured by Western blot quantifying apoptotic proteins Bad/Bcl-2 ratio in U87MG GSCs (**C**) and C6 GSCs (**D**). (**E**) Flow cytometry of Annexin V and Propidium Iodide (PI) apoptotic assays in C6 cells. The graph represents the percentage of positive apoptotic cells. Graphs represent the mean ± S.D. * *p* < 0.05 and *** *p* < 0.001 versus vehicle. ### *p* < 0.001 versus FK506. ψψψ *p* < 0.001 versus Vc. *n* = 4.

To evaluate the effect of FK506 as a chemo-sensitizing agent for GSCs in vitro, the antitumoral drug Vc, a substrate of MRP1 [3], was tested in cell viability and apoptosis assays. Cells were incubated with FK506 (15 ng/mL) and/or Vc (0.1 μM) for 24 h. Treatment with FK506 did not affect cell viability measured by MTT on non-GSCs and GSCs in U87MG and C6 cell lines (Figure 3A,B). Vc treatment decreased cell viability up to 23% only in U87MG non-GSCs (Figure 3A), but Vc in combination with FK506 decreased cell viability in both non-GSCs and GSCs of U87MG and C6 up to ~40% (Figure 3A,B), suggesting a chemo-sensitization effect of FK506. To complement these results, trypan blue exclusion staining assay was performed in U87MG (Figure S1A) and C6 (Figure S1B) GSCs under FK506 and/or Vc treatment. A decrease in cell viability using FK506 alone and associated with Vc was observed in both U87MG and C6 GSCs (Figure S1). To evaluate apoptosis, Bcl-2 (anti-apoptotic) and Bad (pro-apoptotic) protein ratio was measured by Western blot in U87MG GSCs (Figure 3C) and C6 GSCs (D) treated with FK506 and/or Vc [3,20]. A decrease in the Bcl-2/Bad ratio was observed under FK506/Vc treatment (Figure 3C,D). Similarly, an Annexin V/Propidium Iodide staining assay demonstrated that Vc increases the apoptotic C6 GSCs population up to 18% (Figure 3E). Additionally, the combination of FK506 with Vc increased the percentage of apoptotic C6 GSCs up to 21% (Figure 3E). Finally, cleaved caspase-3 was evaluated in U87MG GSCs, and was found to increase in the FK506 treatment in a dose-dependent manner (Figure S2A). The cleaved caspase-3 increased 2.6- and 3.2-fold when using the Vc and FK506/Vc treatments, respectively (Figure S2B). These results suggest that FK506 is able to induce a cytotoxic/pro-apoptotic effect and revert the chemo-resistance in GSCs with an antitumoral drug substrate of MRP1.

### 2.3. FK506 Promotes MRP1-Dependent Chemo-Sensitization In Vivo to Vincristine in GSC-Derived Tumors

**Figure 4:**
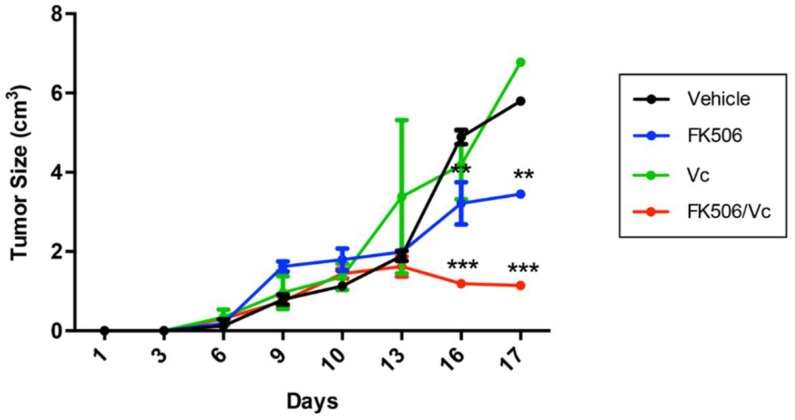
Vincristine in co-treatment with FK506 decreases tumor growth in vivo. A subcutaneous tumor was generated in Sprague-Dawley rats with C6-derived GSCs. Ten days post-inoculation, rats were treated with vehicle (black), FK506 (blue; 2.25 mg/kg/72 h/intravenous), Vc (green; 0.3 mg/kg/72 h/intravenous) or FK506/Vc (red) for seven days. The graph represents tumor volume (in cm^3^) over a total of 17 days. Graphs represent the mean ± S.D. ** *p* < 0.01 and *** *p* < 0.001 versus vehicle. *n* = 4.

**Figure 5:**
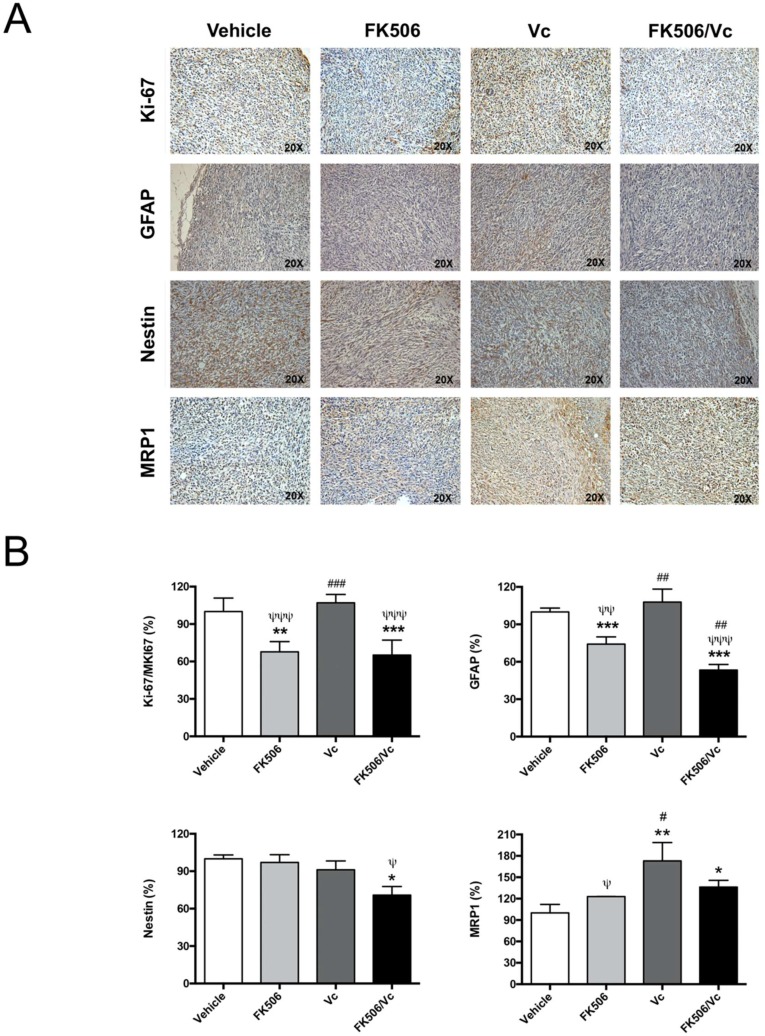
Vincristine in co-treatment with FK506 decreases tumor markers in vivo. (**A**) An immunohistochemistry assay was performed with tumor samples obtained after seven days of treatment (see Figure 4) against Ki-67, Glial Fibrillary Acidic Protein (GFAP), nestin, and MRP1; (**B**) graphs represent the quantification of A as a percentage of expression. Graphs represent the mean ± S.D. * *p* < 0.05; ** *p* < 0.01 and *** *p* < 0.001 versus vehicle. # *p* < 0.05; ## *p* < 0.01 and ### *p* < 0.001 versus FK506. ψ *p* < 0.05; ψψ *p* < 0.01 and ψψψ *p* < 0.001 versus Vc. *n* = 4.

The in vivo chemo-sensitizing effect of FK506 was evaluated using an allogeneic model of a GSC-derived subcutaneous tumor in Sprague-Dawley rats [21]. At day 10 post-GSC inoculation, animals were treated with FK506 and/or Vc for seven days. The tumor size was measured every three days and immunohistopathological analysis was performed at day 17 post-GSC inoculation. Animals treated with FK506 and FK506/Vc combination had smaller tumors compared to those treated with Vc either alone or with a vehicle (Figure 4). Treatment with FK506 alone reduced tumor growth by up to 4 times compared to the vehicle-treated group (Figure 4). Animals treated with the FK506/Vc combination stopped tumor growth, resulting in these being up to ~2- and ~5-fold smaller than the tumors of the Vc- or vehicle-treated groups, respectively (Figure 4). Immunohistopathological analysis revealed that treatment with FK506 and FK506/Vc decreased the expression of Ki-67, a marker of cell proliferation [22], by 33% and 35%, respectively (Figure 5). In turn, the expression of Glial Fibrillary Acidic Protein (GFAP) as a marker of differentiated astroglial cells, and nestin as a marker of neuronal stem cells [3], was evaluated in the tumor samples of treated animals. It was observed that under Vc treatment, GFAP did not change its expression, whereas under FK506 and FK506/Vc treatments GFAP levels decreased by 26% and 47%, respectively (Figure 5). In the case of nestin, no differences in expression were observed under the Vc and FK506 treatments, but a decreased expression was observed in the FK506/Vc combination treatment (Figure 5). As expected, MRP1 transporter expression did not change in treatment with FK506 (Figure 5); however, in Vc and FK506/Vc treatments, MRP1 expression increased up to 73% and 36%, respectively (Figure 5). These results suggest that FK506 is able to chemo-sensitize GSC-derived subcutaneous tumors to Vc treatment in vivo though MRP1 downregulation.

## 3. Discussion

GBM is the most aggressive glioma, characterized by its chemo-resistant phenotype given by a subpopulation of cells with stem-cell like properties, which express transporters that extrude drugs to the extracellular medium. Because of this, studies have focused on reducing the expression and activity of these transporters. For example, the drug FK506 which has shown a chemo-sensitizing effect due to its ability to modulate the accumulation of certain chemo-therapeutic drug substrates of P-glycoprotein (P-gp), Breast Cancer Resistance Protein (BCRP), and MRP1 [19,23]. Previously, we demonstrated that the use of FK506 downregulates the expression and activity of MRP1 in T98 non-GSCs [19]. However, in this study a similar effect was observed for the first time in GSCs, a very important finding considering that GSCs are the main responsible cells for tumor recurrence and chemo-resistance [16]. Here we demonstrate the chemo-sensitizing effect of FK506 on GSCs in vitro and in vivo through the modulation of the expression/activity of MRP1; we even use subtherapeutic concentrations, which would make it easier to scale up to preclinical studies. Nevertheless, in vitro no differences were observed in MRP1 transcript and total protein levels under different concentrations of FK506, neither in GSCs nor non-GSCs. However, it was also observed that MRP1 activity decreased in treatments with 15 ng/mL FK506 for GSCs and 7 ng/mL for non-GSCs in vitro, suggesting that cells with stem-cell like properties have a higher resistance to the action of this drug, maybe due to the higher MRPs levels. At 15 ng/ml of FK506 alone, MRP1 activity decreases but does not inhibit cell proliferation. This can be explained by the decreased MRP1 levels that are not enough to induce cell death in vitro, unlike the treatment in combination with Vc where cells become sensitive. However, in in vitro assays in T98 non-GSCs it was demonstrated that FK506 decreased the mRNA and protein expression of MRP1 [19], but in this article we worked with U87MG and C6 cells, suggesting that the effect could be dependent on the cell line. Despite this, in T98, U87MG, and C6 cells the activity of MRP1 decreases with FK506, which is what matters the most for the chemo-sensitizing effect rather than its protein levels. However, the decrease in MRP1 activity caused by FK506 in U87MG GSCs was greater than that in C6 GSCs, possibly because U87MG cells are O6-methylguanine-DNA methyltransferase (MGMT)-negative and C6 cells are MGMT-positive. We suggest that the presence of MGMT in C6 cells is affecting MRP1 function, because the hypermethylation induced by MGMT can inactivate the expression of several genes and could in this case inactivate a negative regulator of MRP1 or another MRP, so its inhibition with FK506 may not be enough to significantly reduce its activity. It was described that FK506, like Ciclosporin A and Verapamil, interacts with the P-gp transporter binding site, inhibiting its activity [24,25]. This suggests that something similar could be happening with MRP1 regulation. It was also evidenced that FK506 inhibits BCRP transporter in MCF-7 cells, displacing the iodoarylazidoprazosin (IAAP) substrate binding [26]. On the other hand, it is suggested that immunophilins as FKBP (FK506 binding protein) could act as modulators of the ABC transporters, specifically preventing the interaction between FKBP12 and P-gp [27,28]. FK506 reduces MRP1 activity by almost half, but cell death does not increase significantly in cells treated with FK506 as a Vc chemo-sensitizer. These results suggest that the effect could be MRP1-independent, because U87MG GSCs express other multiple resistance-associated proteins (MRPs), such as multiple resistance-associated protein-3 (MRP3). MRPs are very similar and have specific substrates, as Teniposide for MRP3 and Doxorubicine for MRP1, while also sharing substrates as Vc, which can also be extruded by MRP3 to a lesser extent [15]. This could further induce MRP3 expression and activity as a compensatory effect when MRP1 is downregulated. Despite the low level of viability, it is observed that FK506 is able to activate caspase-3, which suggests that the effect could be independent of MRP1 through FKBP, since it has been demonstrated that the binding of FK506 to FKBP promotes oligomerization with pro-caspases, thus promoting its activation [29]. However, this has not been proven for GSCs and it would be an interesting subject for study. Despite these mechanisms, FK506 has a dual role as an immunosuppressant, which can be complicated considering that it is not advisable to decrease the immune response against the tumor in cancer patients. However, the mechanism of action of this drug inhibits FKBP by decreasing the levels of IL-2, thereby decreasing the inflammatory microenvironment and the immune response in the tumor [5]. In this context, this effect could be counterproductive since the immune response is of major importance for the outcome of the disease; however, the inflammatory microenvironment that can promote the expression of MRPs and grow factors through the overproduction of adenosine [16] is even more important. Despite these effects, FK506 concentrations used in this article are at subtherapeutic levels, which have not been shown to have an immunosuppressive effect [19]. Despite these possible mechanisms, in the in vivo tests we observed changes in MRP1 expression in treatment with FK506, so it is possible that the lower transporter activity is due to a decreased protein expression. It has been described that treatments with FK506 and Vc decrease cell viability by enhancing apoptosis [30,31]. These results suggest that FK506 has a chemo-sensitizing effect, which had already been tested in different cancer cell lines, including GBM, but not in GSCs [19,23,32]. In this study, the in vivo assay demonstrates that treatment with FK506 and Vc decrease MRP1 levels, compared to Vc treatment alone. MRP1 levels induced by Vc have been previously described in different cell lines, suggesting a resistance mechanism [3,33,34]. FK506 is capable of reversing this mechanism, as the treatment of FK506 in combination with Vc showed, reducing tumor volume as well as reducing GFAP, nestin, and Ki67 levels, which are associated with GBM aggressiveness [35,36]. We proposed that FK506 and Vc can act synergistically because FK506 potentiates the antitumor effect of Vc. Vc decreases proliferation by itself in different cellular models; however, if cells express high levels of MRP1, as GSCs do, Vc may not decrease cell viability by itself and can even induce MRP1 expression. In addition, the combined treatment with FK506 could reverse this effect by decreasing the levels of MRP1, killing the cells and also potentiating the effect of Vc when there is no transporter that extrudes it to the extracellular medium. On the other hand, in vivo treatment with FK506 alone decreased the tumor size. Yet, we suggest that it was not due to the decrease in MRP1, since MRP1 would not influence tumor development by itself, but only in combination with a substrate antitumoral drug such as Vc. We propose that the effect of FK506 alone decreased tumor size because it is an immunosuppressant that diminishes the inflammatory microenvironment within the tumor and decreases the concentration of interleukins that promote tumor growth. Because the mechanism of action by which FK506 inhibits the activity of MRP1 is unknown, we propose that the effects produced by FK506 could be due to a direct link to the transporter, acting as a competitive inhibitor, or through binding to FKBP, preventing MRP1 translocation to the cell membrane. Finally, as a projection, it would be recommendable to perform intrathecal models because subcutaneous models present some disadvantages, mainly concerning the cerebral microenvironment and the absence of the blood-brain barrier. This is a relevant factor in the action of some drugs, because several antitumoral drugs such as Vc present a poor penetration to the blood-brain barrier due to the action of P-gp [37,38]. Despite this, with the subcutaneous model, we demonstrated the chemo-sensitizing effect of FK506 on the treatment of GSC-derived tumors using Vc.

## 4. Materials and Methods

### 4.1. Cell Culture

Human U87MG GBM (ATCC, HTB-14TM) and rat C6 glioma (ATCC, CCL-107TM) cell lines were grown in differentiation (DMEM-F12 medium supplemented with 10% fetal bovine serum and penicillin-streptomycin (Life Technologies, Carlsbad, CA, USA)) or Neurosphere (Neurobasal medium supplemented with 20 ng/mL basic Fibroblast Growth Factor (bFGF), 20 ng/mL Epidermal Growth Factor (EGF), Glutamax 1×, B-27 1×, all purchased from Gibco^®^ (Thermo Fisher Scientific Inc., Waltham, MA, USA)) media for 7–10 days to generate non-GSCs and GSCs, respectively (Figure S3) [21].

### 4.2. RT-qPCR

mRNA levels of MRP1 in non-GSCs and GSCs of U87MG and C6 cell lines were measured by RT-qPCR using the ΔΔ*C*t method [21]. One μg of total RNA was reverse transcribed using the Tetro cDNA Synthesis kit (Bioline Reagents Limited, London, UK). For qPCR reaction, 250 nM of each primer (Table S1) was mixed using the 5× HOT FIREPol^®^ EvaGreen^®^ qPCR Mix Plus (ROX) kit (Solis BioDyne, Tartu, Estonia) as previously described [3]. All qPCRs were conducted in triplicate for each treatment, with their respective dissociation curves, using actin beta (ACTB) mRNA levels as a normalizer gene.

### 4.3. Immunocytofluorescence

Non-GSCs and GSCs of U87MG and C6 cell lines were grown on circular coverslips at semi-confluence, and GSCs were adhered to coverslips using Poly-l-Lysine for 30 min, as previously described by Torres et al. [3]. Then cells were washed with 0.1 M phosphate buffer (pH 7.4), fixed with paraformaldehyde 4% for 10 min, and permeabilized using 0.3% Triton X-100 in 1× Phosphate Buffered Saline (PBS) for 15 min. Preparations were blocked with 2.5% normal horse serum and incubated with anti-CD44 (#3570, Cell Signaling Technology, Inc., Beverly, MA, United States) and anti-nestin (sc-33677, Santa Cruz Biotechnology, Dallas, Texas, United States) antibodies. Finally, DAPI staining was used as a counterstain and cells were visualized using an epifluorescence microscope (Zeiss, Jena, Germany).

### 4.4. Flow Cytometry

MRP1-positive cells were measured by flow cytometry (FACS Jazz; BD Biosciences, Franklin Lakes, NJ, USA) using the protocol previously described by Torres et al. [3]. Briefly, non-GSCs and GSCs were treated with vehicle (1× PBS) and FK506 (7, 15, and 30 ng/mL) (Cat. No. 3631; Tocris Bioscience, Bristol, UK) for 24 h. Cells were fixed with PFA (paraformaldehyde) 3.7%, blocked (1× PBS-BSA (bovine serum albumin) 0.5% at room temperature) and marked with anti-MRP1 (sc-18835, Santa Cruz Biotechnology) antibody followed by a secondary anti-mouse Alexa 488 (Life Technologies). Lastly, events were acquired through the FL1 filter of the cytometer.

### 4.5. CFDA-Retention Assay

To evaluate the MRP1 activity we used the CDFA-retention assay previously described by Torres et al. [3]. Briefly, non-GSCs and GSCs were exposed to vehicle (1× PBS), FK506 (7, 15, and 30 ng/mL) and/or Vc (0,1 µM) for 24 h at 37 °C in 24-well plates. Cells were loaded with CFDA for 15 min, washed three times with 1× PBS, and then incubated for 15 min in serum-free DMEM/F-12 medium at 37 °C. Finally, cells were washed three times with ice-cold 1× PBS, lysed (1× PBS-0.4% Triton X-100), and the CFDA accumulation was measured (exciting at 488 nm and collecting emission at 530 nm) using a spectrofluorometer (PERK1/2in–Elmer), with values normalized to the total protein content of lysed cells.

### 4.6. MTT Assay

To evaluate the cytotoxic effect of FK506 and/or Vc treatment, the MTT assay described by Garrido et al. [19] was performed. Cells were grown in 96-well plates (10^5^ cells in 50 µL of DMEM-F12 or Neurobasal medium per well) for 24 h and exposed to FK506 (15 ng/mL) and/or Vc (0,1 µM) for 24 h. Then 50 µL of MTT reagent (5 mg/mL) was added per well and the mixture was incubated for 1 h at 37 °C. Formazan crystals were dissolved by adding 100 μL of lysis buffer (10% SDS (sodium dodecyl sulfate), 45% dimethylformamide, pH 4.5) overnight at 37 °C. Finally, absorbance was measured at 550 nm (Synergy HT, BioTek Instruments, Inc., Winooski, VT, USA) and values were expressed as a percentage using vehicle-treated cells as a calibrator.

### 4.7. Trypan Blue Staining Assay

A number of 10,000 U87MG or C6 GSCs per well were seeded in a 96-wells plate and incubated with FK506 (15 ng/mL) and/or Vc (0,1 µM) for 24 h at 37 °C. Ten microliters of cell suspension was mixed with 10 µL of 0.4% trypan blue (T10282, Thermo Fisher Scientific Inc., Waltham, MA, USA) and a quantification of cell viability was performed using an automated cell counter (C10227, Thermo Fisher Scientific Inc., Waltham, MA, USA). Percentages of cell viabilities were graphed (GraphPad Prism^®^ 6.01, La Jolla, CA, USA) using the vehicle-treated group as a calibrator.

### 4.8. Western Blot

Total protein extracts (50 μg) of U87MG GSCs and C6 GSCs treated with FK506 and/or Vc were separated by 8% and 15% SDS-PAGE, followed by the transfer to 0.22-μm PVDF (polyvinylidene fluoride) membranes (Bio-Rad, Hercules, CA, USA). Membranes were blocked using 5% non-fat milk for 45 min and then incubated with anti-CD44 (#3570, Cell Signaling Technology, Inc., Beverly, MA, United States), anti-nestin (sc-33677, Santa Cruz Biotechnology, Dallas, Texas, United States), anti-cleaved caspase-3 (#9661, Cell Signaling Technology, Inc., Beverly, MA, United States), anti-Bcl-2 (MAB8272, R&D Systems, Inc. Minneapolis, United States ), and anti-Bad (MAB6405, R&D Systems, Inc. Minneapolis, United Stated) antibodies overnight. Finally, membranes were incubated with a secondary HRP (Horseradish Peroxidase)-conjugated IgG antibody (DAKO Agilent, Santa Clara, CA, United States) for 1 h at room temperature, and detected using SuperSignal^TM^ West Dura (Thermo Fisher Scientific Inc., Waltham, MA, United States) in the image analysis system syngene G: Box (Synoptics Ltd., Cambridge, UK). The images were analyzed by densitometry (Image J software, 1.52a, NIH, Bethesda, MD, United States) and normalized by β-actin (sc-47778-HRP, Santa Cruz Biotechnology) expression using vehicle-treated (1× PBS) cells as a calibrator.

### 4.9. Annexin-V/Propidium Iodide Assay

A quantity of 1 × 10^6^ of U87MG GSCs was treated with vehicle (1× PBS), FK506, and/or Vc for 24 h at 37 °C in 6-well plates. Then cells were processed in accordance with the FITC Annexin-V Apoptosis Detection Kit II protocol (BD Pharmingen^TM^, San Jose, CA, United States). Samples were analyzed by FACS Jazz flow cytometry (BD Biosciences, Franklin Lakes, NJ, USA) considering Annexin-V/Propidium Iodide positive-cells as apoptotic GSCs.

### 4.10. Generation of GSC-Derivative Subcutaneous Tumors

Animals were maintained under standard laboratory conditions approved by the Ethics Committee of Animal Experiments at the Universidad Austral de Chile (Permit Number: 248-2016; date: 23 March 2016). A total of 16 male Sprague-Dawley rats (200–250 g) previously anesthetized (ketamine (100 mg/kg)/xylazine (10 mg/kg) intraperitoneal) were inoculated with 2 × 10^5^ C6 GSCs via subcutaneous injection. At day 10 post-inoculation, rats were divided in four groups (i) 1× PBS (Vehicle), (ii) FK506 (2.25 mg/kg/72 h/intravenous), (iii) Vc (0.3 mg/kg/72 h/intravenous), and (iv) FK506/Vc, and were treated for seven days. Tumor size was measured every three days until day 17 post-inoculation when rats were euthanized by Sodium Thiopental administration (120 mg/kg/intraperitoneal).

### 4.11. Immunohystochemistry

Seventeen days post-inoculation, C6 GSC-derived subcutaneous tumors were removed, fixed in 3.7% paraformaldehyde (Sigma-Aldrich, Darmstadt, Germany), dewaxed with xylol, and rehydrated using alcohols in decreasing concentration. Paraffinated tumors were sectioned (5 μm) and mounted on silanized slides. Then, samples were dewaxed with xylol, rehydrated using alcohols in decreasing concentration, and immersed in heated citrate buffer (pH 6) for antigen retrieval. Endogenous peroxidase was blocked using 3% hydrogen peroxide, followed by incubation with 5% BSA for 1 h and incubation with anti-MRP1 (sc-18836; Santa Cruz Biotechnology), anti-Ki67 (sc-15402; Santa Cruz Biotechnology), anti-nestin (ab22035, abcam, Cambridge, MA, United States), and anti-GFAP (sc-33673, Santa Cruz Biotechnology) antibodies. Finally, immunodetections were detected using the ImmPRESS HRP Universal Antibody Polymer Detection Kit (Vector Laboratories, Burlingame, CA, United States) with Liquid DAB + Substrate Chromogen System, K3468, DAKO (Santa Clara, CA, United States) following the manufacturer′s instructions and using hematoxilin as a counterstain. Samples were visualized using a light microscope (Zeiss) and 10 images (20×) were processed using ImageJ software (NIH), considering the values of vehicle treatment as a calibrator, and normalized to 100%.

### 4.12. Graphs and Statistics

GraphPad Prism^®^ (6.01, La Jolla, CA, USA) software was used to make graphs and statistics. Values were means ± Standard Deviation (S.D.) and statistical analysis was carried out on raw data using ANOVA, Student’s *t*-test (unpaired data), and Tukey test. A *p* value < 0.05 was considered statistically significant.

## 5. Conclusions

In this study, a cytotoxic, pro-apoptotic, and chemo-sensitizing effect of FK506 through MRP1 downregulation in GSCs was shown for the first time. Previously, its effect had been demonstrated for non-GSCs. However, due to the importance of the stem-like cell subpopulation, it is necessary to find drugs that can decrease chemo-resistance in most subpopulations within the tumor. Despite this, FK506 was not able to decrease MRP1 transcript and protein levels in vitro in GSCs; however, the transporter activity decreased, and the cells became more susceptible to treatment with Vc, inducing apoptosis. On the other hand, in vivo tests in allogeneic models showed that tumors derived from GSCs and treated with FK506 were more sensitive to Vc treatment, as immunohistochemistry showed that the levels of MRP1 decreased in vivo. These results suggest that the chemo-sensitizing effect of FK506 in vivo is produced by the downregulation of MRP1 levels, which finally decreases its activity of extruding Vc to the extracellular medium. All of these results support the idea that many of the current treatments that are ineffective can be complemented with chemo-sensitizing drugs such as FK506.

## Supplementary Materials

Supplementary materials can be found at http://www.mdpi.com/1422-0067/19/9/2697/s1.
