# Supplementary material for: FK506 Attenuates the MRP1-Mediated Chemoresistant Phenotype in Glioblastoma Stem-Like Cells
Source: Int J Mol Sci. 2018 Sep 11;19(9):2697. doi: 10.3390/ijms19092697 (PMC6164673; doi:10.3390/ijms19092697)
Supplement: Supplementary file 1 [file ijms-19-02697-s001.pdf]

FK506 Attenuates the MRP1-Mediated Chemo-Resistant Phenotype in Glioblastoma Stem-Like Cells

Ángelo Torres, Valentina Arriagada, José Ignacio Erices, María de los Ángeles Toro, José Dellis Rocha, Ignacio Niechi, Cristian Carrasco, Carlos Oyarzún and Claudia Quezada

Table S1

List of primers used for RT-qPCR

Gene	Forward primer	Reverse primer
**MRP1 human**	5' GGACTTTCGTGTGCTCCTGA 3'	5' AGGTCAAGCTTTCCGTGTACTG 3'
**MRP1 rat**	5’ TGAACCATGAGTGTGCAGAA 3’	5’ TCACACCAAGCCAGCATCCT 3’
**ACTB human**	5' GGGGTCTTGAAGGTCTCA 3'	5' TGTCACCAACTGGGACGA 3'
**ACTB rat**	5’ TGTCACCAACTGGGACGATA 3’	5’ GGGGTCTTGAAGGTCTCAAA 3’

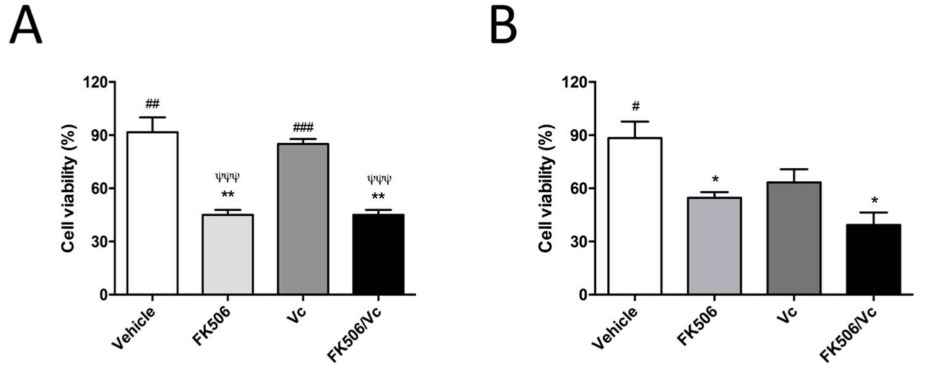

Figure S1. Cell viability measured by trypan blue exclusion assay in U87MG (A) and C6 (B) GSCs treated with vehicle (DMSO), FK506 (15 ng/mL) and/or Vincristine (Vc; 0,1 μM). The graph represents the percentage of cell viability using vehicle as calibrator. Graphs represent the mean ± S.D. *P < 0.05 and **P < 0.01 versus vehicle. #P < 0.05, ##P < 0.01 and ###P < 0.001 versus FK506. ψψψ P < 0.001 versus Vc. n = 3.

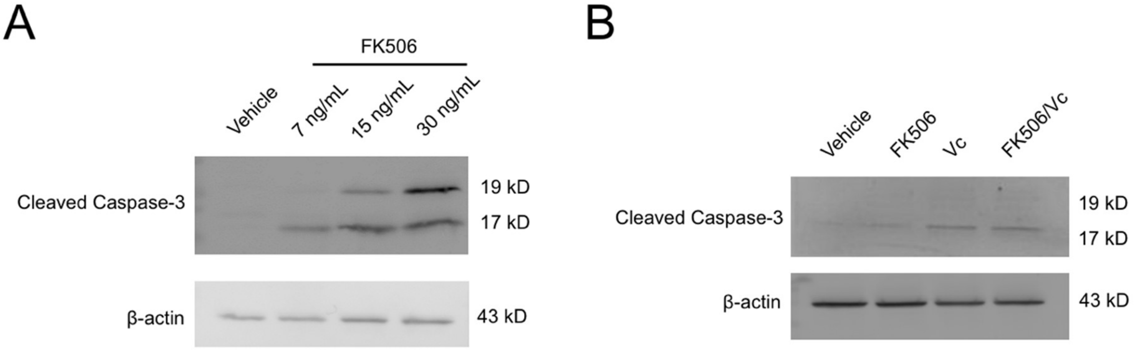

Figure S2. A Cleaved caspase-3 in U87MG-GSCs treated with vehicle (DMSO) and FK506 (7, 15 and 30 ng/mL). **B** Cleaved caspase-3 in C6-GSCs treated with vehicle (DMSO), FK506 (15 ng/mL) and/or Vincristine (Vc; 0,1 μM). β-actin expression was used as normalizer in A) and B).

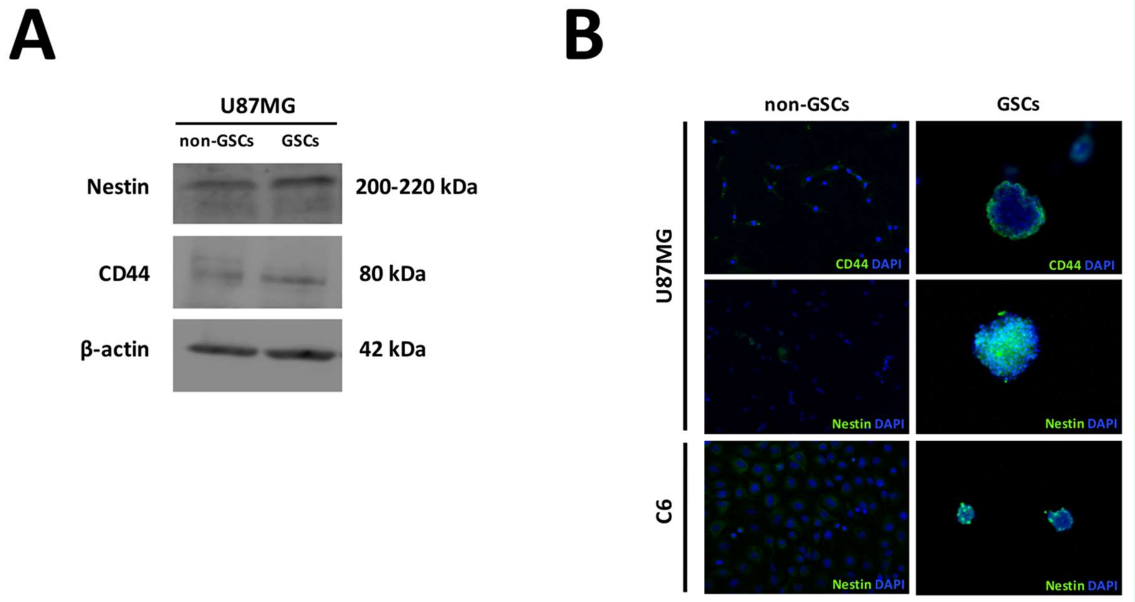

Figure S3. Stem cells markers expression in non-GSCs and GSCs of U87MG and C6 cell lines. **A** Expression of Nestin and CD44 in U87MG non-GSCs and GSCs by western blot. β-actin expression was used as normalizer. **B** Immunocytofluorescence of CD44 and Nestin in non-GSCs and GSCs of U87MG and C6 cell lines. A secondary antibody Alexa 488 (green) was used to detect CD44 and Nestin expression. DAPI (blue) stain was used as counterstain of nuclei. Magnification 200x.
